# Editorial: How to Play the Science Game: Insights on Scientific Teams

**DOI:** 10.3389/frma.2021.802557

**Published:** 2021-11-29

**Authors:** Stijn Kelchtermans, Cindy Lopes-Bento, Fabiana Visentin, Cornelia Lawson

**Affiliations:** ^1^ Department of Management, Strategy and Innovation, KU Leuven, Leuven, Belgium; ^2^ Department of Marketing and Strategy, University of Hasselt, Hasselt, Belgium; ^3^ Fonds National de la Recherche, Luxembourg, Luxembourg; ^4^ School of Business and Economics, Maastricht University, Maastricht, Netherlands; ^5^ United Nations University—Maastricht Economic and Social Research Institute on Innovation and Technology, Maastricht, Netherlands; ^6^ Alliance Manchester Business School, Faculty of Humanities, The University of Manchester, Manchester, United Kingdom

**Keywords:** teams and team work, science, authorship, research funding, entrepreneurial academic teams, societal impact

Recent research has noted the increasing importance of teams for conducting science (Wuchty et al., 2007; Stephan, 2012). An often-cited rationale for the growing reliance on teamwork in science is the “burden of knowledge” i.e., the ever-accumulating knowledge base that must be mastered to push the scientific frontier forward (Jones, 2009). Given their growing importance, recent studies have investigated scientific teams from various perspectives, learning and productivity effects that take place among team members (Ayoubi et al., 2017), or the organization of research and division of tasks within teams (Haeussler and Sauermann, 2020). Despite this progress, a better understanding of the role and purpose of teams and how they affect the organization of science is crucial. The articles published as part of this Research Topic further expand our understanding of the mechanisms at work in scientific teams and their relation to the context in which scientists operate. The collection of articles addresses the topic in diverse settings, using complementary perspectives and methods.

Pruschak provides new insights on the inner workings of teams, zooming in on a key mechanism that underlies the allocation of credit in the science system: authorship. In a survey of social scientists, he shows that teams routinely resort to division of labour among team members, and (implicitly or explicitly) apply the Vancouver criteria for making decisions about co-authorship. Against the backdrop of the growing importance of big data also in the social sciences, the study reveals that data work is a research task that is not associated with an increased chance of recognition as a co-author in the social sciences. The author calls on journal publishers and editors to introduce clear authorship guidelines, and institutions to more formally recognize data efforts in hiring and promotion decisions.

Turning attention towards teams’ external relations, Fecher et al. study transdisciplinary teams involving scientists in the Social Sciences and Humanities (SSH) and societal stakeholders. Using a survey of 125 experts in Germany, they identify key issues in the interaction between academics and societal stakeholders. The framework they develop highlights how SSH scientists not only encounter difficulties in effectively translating findings to a broader audience, but also cope with a lack of institutional incentives, resources and support as well as epistemic challenges both within SSH disciplines and vis-à-vis the natural sciences. Also, the uptake of SSH expertise by societal stakeholders is non-trivial and would benefit from more thoughtful “expectation management” by scientists and societal stakeholders. The article raises pertinent questions about how the societal impact of SSH should be understood in the first place and how SSH disciplines—and society as a whole—could benefit from more formative rather than merely quantitative assessments. In doing so it highlights the challenges of cross-sector collaboration.

A second article addressing external linkages of scientific teams by Öcalan-Özel and Llerena studies the relation between teams’ industry collaborations and their success in securing research funding. They use unique data on applications to the European Collaboration Research (EUROSCORES), a pan-European funding program with a three-stage evaluation schema. By analysing 1,642 outline proposals submitted to the first stage, and 886 full proposals reaching the final stage in 2002–2010, Öcalan-Özel and Llerena distinguish industry partners’ direct and indirect engagement in applicants’ teams. It seems that evaluators use different selection criteria across the selection stages. While an industrial partner in the team (direct engagement) is associated with a higher probability of passing the first stage of selection, the other selection stages are not affected by the presence of an industrial partner. Looking at the previous collaborations between the applicants and industrial partners (indirect engagement), the authors do not find a significant association with success in securing research funding. The article opens a debate on the importance and value of the collaborations between industry and universities.

In the final contribution, Clayton and Feldman consider an important impediment to the study of scientific teams. They point at the difficulty of compiling data, which often requires multiple data sources, and the difficulty of differentiating individual and team level characteristics. The data and discussion they present considers how academics form teams to start new companies. To this end, they review the literature on entrepreneurial team formation and dedicate particular attention to data and how data may be collected to study entrepreneurial academic teams. Analyzing life science entrepreneurs in North Carolina’s Research Triangle region, relying on data compiled from over 30 different sources, Clayton and Feldman pay particular attention to the various configurations of such teams and their implication for firm behavior with regards to survival, firm growth and patenting. They conclude that teams involving academics and individuals with an industry background may be better placed to succeed. The article finally presents several challenges for empirical analyses on research on academic teams and offers a guidepost for future research on the topic.

The four articles in this topic reveal the importance and challenges of task diversity for teams. A major contribution of this topic is highlighting the role of non-academic actors that join scientific teams and the dynamics of teams from social science domains. A second contribution is the presentation of diverse methodologies and metrics to study scientific teams. The use of administrative data as demonstrated in the last two studies is very promising but also highlights remaining limitations to map teams comprehensively.
